# Risk patterns of lung cancer mortality in northern Thailand

**DOI:** 10.1186/s12889-018-6025-1

**Published:** 2018-09-24

**Authors:** Apinut Rankantha, Imjai Chitapanarux, Donsuk Pongnikorn, Sukon Prasitwattanaseree, Walaithip Bunyatisai, Patumrat Sripan, Patrinee Traisathit

**Affiliations:** 1 Graduate School, Chiang Mai University, Chiang Mai, Thailand; 2 Department of Statistics, Faculty of Science, Chiang Mai University, Chiang Mai, Thailand; 3 Division of Radiation Oncology, Department of Radiology, Faculty of Medicine, Chiang Mai University, Chiang Mai, Thailand; 4 Chiang Mai Cancer Registry, Maharaj Nakorn Chiang Mai Hospital, Faculty of Medicine, Chiang Mai University, Chiang Mai, Thailand; 5 Northern Thai Research Group of Radiation Oncology (NTRG-RO), Faculty of Medicine, Chiang Mai University, Chiang Mai, Thailand; 6 Cancer Registry Unit, Lampang Cancer Hospital, Lampang, Thailand; 7 Center of Excellence in Bioresources for Agriculture, Industry and Medicine, Faculty of Science, Chiang Mai University, Chiang Mai, Thailand

**Keywords:** Lung cancer, Mortality, Relative risk, Besag-York-Mollié

## Abstract

**Background:**

Over the past decade, lung cancers have exhibited a disproportionately high mortality and increasing mortality trend in Thailand, especially in the northern region, and prevention strategies have consequently become more important in this region. Spatial analysis studies may be helpful in guiding any strategy put in place to respond to the risk of lung cancer mortality in specific areas. The aim of our study was to identify risk patterns for lung cancer mortality within the northern region of Thailand.

**Methods:**

In the spatial analysis, the relative risk (RR) was used as a measure of the risk of lung cancer mortality in 81 districts of northern Thailand between 2008 and 2017. The RR was estimated according to the Besag-York-Mollié autoregressive spatial model performed using the OpenBUGS routine in the R statistical software package. We presented the overall and gender specific lung cancer mortality risk patterns of the region using the Quantum Geographic Information System.

**Results:**

The overall risk of lung cancer mortality was the highest in the west of northern Thailand, especially in the Hang Dong, Doi Lo, and San Pa Tong districts. For both genders, the risk patterns of lung cancer mortality indicated a high risk in the west of northern Thailand, with females being at a higher risk than males.

**Conclusions:**

There was distinct geographical variation in risk patterns of lung cancer mortality in Thailand. Differences could be related to differences in risk factors such as ground-based radon and air pollution. This study provides a starting point for estimating the spatial pattern of the risk of lung cancer mortality and for examining associations between geographic risk factors and lung mortality for further studies.

**Electronic supplementary material:**

The online version of this article (10.1186/s12889-018-6025-1) contains supplementary material, which is available to authorized users.

## Background

Lung cancer is the leading cause of cancer-related mortality worldwide, and from 1990 to 2015, it was the most common cause of cancer mortality in 113 countries [1–3]. Thailand is one of those countries in which lung cancer has been the leading cause of mortality and healthcare burden compared to other cancer types, especially in the northern region [4–6]. Of all the regions in Thailand, the northern part ranks first in mortality rate caused by lung cancer and in addition, has the highest mortality rate compared to other types of cancer [4, 5, 7].

Over the past few decades, a considerable number of studies have been conducted to investigate factors associated with the occurrence, treatment, and outcomes of lung cancer in northern Thailand, such as demographic characteristics (including hereditary genetic mutations, gender, and geographic location), environmental hazards (such as exposure to indoor radon, smoke, and air pollution), patient health status and behavior, and healthcare providers’ characteristics [8–17]. All of these factors might be contributing to the variations in cancer incidence, diagnosis, and outcomes [18]. Measuring the spatial distributions of diseases could help describe the possible determinants of disease occurrence or the outcome of healthcare interventions such as the aforementioned factors [19–21]. Moreover, spatial incidence patterns have been used to explore injuries and non-communicable disease incidences as these conditions are the result of interactions between behaviors, lifestyles, and the environment related to residential area and geographical differences [22–26].

Focusing on Chiang Mai province, the high-risk districts have a problem with high air pollution from particulate matter with a diameter smaller than 10 μm (PM10) in northern Thailand [16, 27]. Moreover, this area is surrounded by high mountains that block diffusion and redirect airflow, thereby exacerbating air pollution accumulation along the foothills of the mountains [28–30]. In addition, urban development has been linked to the occurrence of lung cancer and leading to death [31, 32]. The findings in [11] indicate that the urban growth in Chiang Mai province has had a tendency to increase over time while air quality has simultaneously declined. In addition, the risk patterns of lung cancer mortality have been found to be different geographically between males and females [26, 33, 34].

Small-area geographic data recommended when studying the magnitude of geographical health inequality [35–37]. In Thailand, small-areas studies have been rare, studies such as Aungkulanon et al. [4] which presented geographical patterns of lung cancer mortality using the standardized mortality ratio (SMR). The SMR represents the ratio of the observed and expected number of lung cancer mortalities for the total population. The SMR is a reliable risk measure for large geographical regions but may be unreliable for small areas [38]. The relative risk (RR) from the well-known Bayesian spatial model may overcome the problem of SMR [39–42].

The aim of this study was to identify risk patterns of lung cancer mortality across 81 districts spanning the six northern provinces of Thailand, specifically Chiang Mai, Chiang Rai, Phrae, Phayao, Lampang, and Lamphun. The method consists of using a well-known Bayesian spatial model to estimate the relative risk at the district level. The analysis was performed for both genders combined, and separately to obtain gender-specific estimates of the relative risk.

## Methods

### The study area

**Fig. 1:**
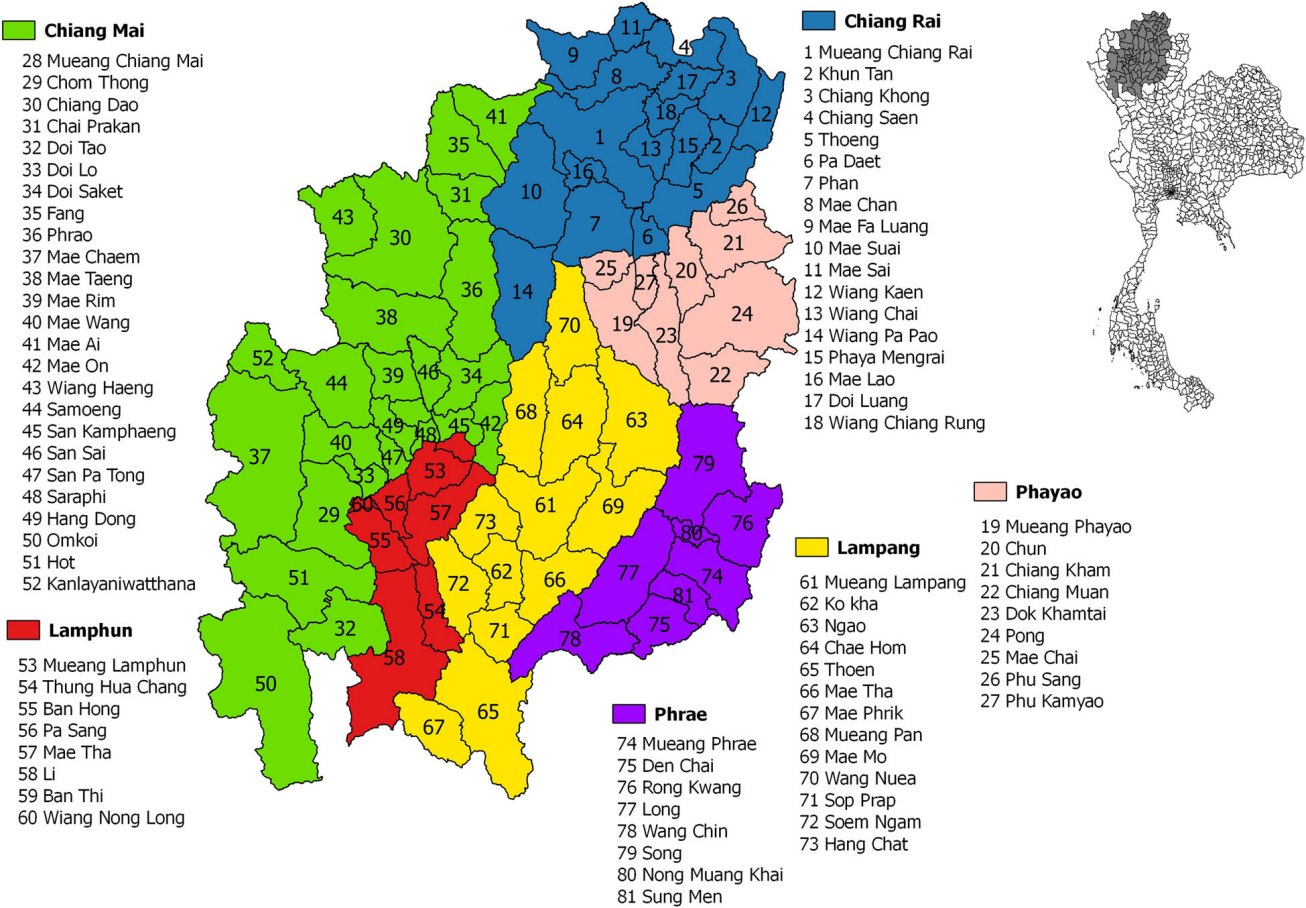
The six northern Thailand provinces determined into 81 districts within Thailand. Source of reference map: United Nations Office for the Coordination of Humanitarian Affairs - Regional Office for Asia and the Pacific [52]

In this registry-based study, we focused on data from 81 districts within 6 provinces of the northern region of Thailand: Chiang Mai, Chiang Rai, Lampang, Lamphun, Phayao, and Phrae (Fig. 1). The median population size of the districts was 50,132 (range 11,025–229,033), of which the numbers of males and females were 24,346 (range 5735–110,167) and 25,843 (range 5290–122,063), respectively.

### Data collection

We used data on lung cancer mortality from the Chiang Mai Cancer Registry, Faculty of Medicine, Chiang Mai University and the Lampang Cancer Registry, Lampang Cancer Hospital, that covered population-based cancer in Northern Thailand. Moreover, data collection was completed adequately and the completeness of the data was satisfactory for our study [43]. The participants in this study were 10,468 adults (> 15 years old) diagnosed with cancer of the lung (coded as International Classification of Diseases version 10 (ICD-10) C33–34) between 1 January 2008 and 31 December 2014 who then subsequently died from any cause between 1 January 2008 to 1 July 2017. The population census database from the Official Statistics Registration Systems, Department of Provincial Administration, Thailand, was used as the denominator for calculating age- and gender-specific mortality rates divided into 18 age groups (0–4, 5–9, ..., 80–84, 85+) and by gender for the study area.

In this study, the observed number of deaths was derived from the number of deaths of people previously diagnosed with lung cancer from any cause during the study period in the 81 districts for the overall spatial analysis. For the spatial analysis by gender, the observed number of deaths by gender in each district was derived in the same way as for the overall spatial analysis. According to the coded data at the district level, the median number of observed cases per district was 100 (range 9–469), of which the median number of males was 57 (range 9–266) and that of females was 39 (range 0–203). The expected number of cases in each district was calculated by taking the specific mortality rates of lung cancer and multiplying the population at the midpoint of the study periods for each district broken down into the same strata. The population data were likewise drawn from the Official Statistics Registration Systems, Department of Provincial Administration, Thailand.

### Statistical analyses

In the spatial analysis, the observed number of deaths *Y*_*i*_ (*i* = 1*,...,*81) are assumed to follow an independent Poisson distribution given *θ*_*i*_*, Y*_*i*_ *~Poisson*(*θ*_*i*_*E*_*i*_), which is the unknown relative risk in each district, and *E*_*i*_, which is the expected number of cases [44]. The Besag-York-Mollié (BYM) model [45] used to estimate the relative risk of lung cancer mortality includes both spatial heterogeneity typically represented using the aggregated neighbors of each district and uncorrelated spatial heterogeneity as follows [42]:

*log*(*θ*_*i*_) = *α* + *u*_*i*_ + *v*_*i*_, where *α* is the intercept of the relative risk, and *u*_*i*_ and *v*_*i*_ are the correlated and uncorrelated heterogeneity components, respectively. *u*_*i*_ is assumed to apply the spatial correlation since the relative risk estimation in each *i* is dependent on the neighboring areas. The spatial structure is defined based on the adjacent neighbors of each district and is assumed to follow a Gaussian intrinsic autoregression. The *v*_*i*_ uncorrelated heterogeneities are assumed to follow a normal distribution with zero mean (see [44, 46] for a more in-depth explanation of this component). This model was used to explore the RR spatial distribution for the risk of lung cancer mortality among the districts. The Gibbs sampling technique was implemented to estimate the parameters from the BYM model with the openBUGS [47] implementation in R syntax [48] presented by Gerber and Furrer [49] [see the R syntax in Additional file 1]. Furthermore, the deviance information criterion (DIC) for the model fitting result was calculated overall and by gender [50]. We estimated the RR of the lung cancer mortality for each district and its 95% credible interval (CrI) with the intercept with the two random effects (spatial heterogeneity and uncorrelated spatial heterogeneity). The map of the risk patterns of lung cancer mortality for the 81 districts in 6 northern Thailand provinces was created using the Quantum Geographic Information System (QGIS) program version 2.8.1 [51]. The map used in this study were public shapefile dataset derived from United Nations Office for the Coordination of Humanitarian Affairs - Regional Office for Asia and the Pacific [52].

## Results

### Characteristics

The lung cancer mortality data during 2008–2017 in the 6 northern Thailand provinces consisted of 10,468 deaths of which 6148 (58%) were male. We found that the largest number of cases occurred in Chiang Mai province (Table 1). The number of observed deaths from lung cancer were higher than the expected number for males in Chiang Mai province and for females in Chiang Rai province.

**Table 1:** The observed and expected number of lung cancer mortality in the six northern Thailand provinces

	Total Population Over 15 Years Old	Overall		Gender			
				Male		Female	
		Obs.	Exp.	Obs.	Exp.	Obs.	Exp.
Chiang Mai	1,294,412	4141	3356	2390	1965	1751	1391
Chiang Rai	935,620	2181	2265	1208	1350	973	915
Lampang	644,750	1635	1811	1026	1063	609	748
Lamphun	343,326	735	963	450	567	285	396
Phrae	390,093	913	1062	580	604	333	458
Phayao	410,391	863	1011	494	599	369	412
Total	4,018,592	10,468	10,468	6148	6148	4320	4320

*Obs* Observed number, *Exp* Expected number

### The overall spatial analysis

**Fig. 2:**
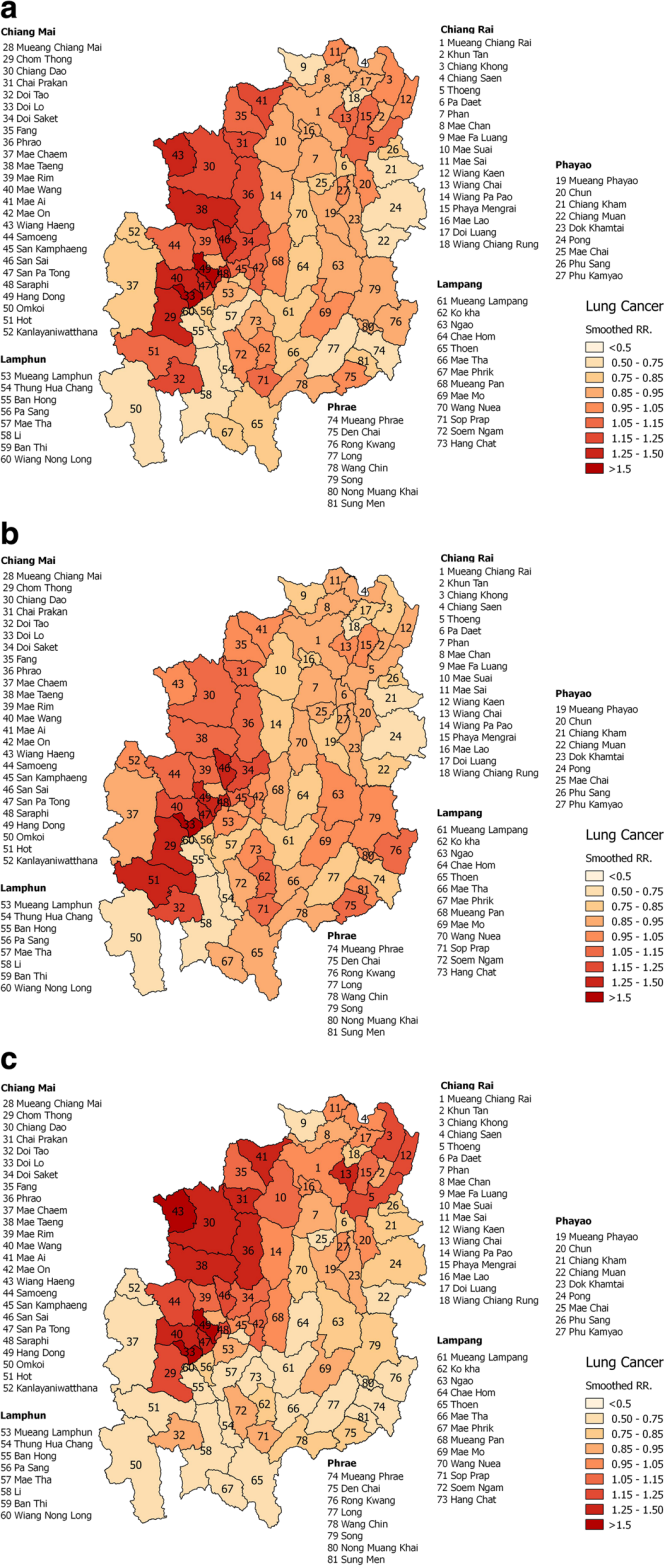
Relative risk patterns of lung cancer mortality in the six northern Thailand provinces: **a** overall, **b** male, and **c** female. Source of reference map: United Nations Office for the Coordination of Humanitarian Affairs - Regional Office for Asia and the Pacific [52]

The overall DIC value from the BYM model was found to be 552.8. The RR of lung cancer mortality over the whole study ranged from 0.581–1.666 (Fig. 2a). The highest RR was found in Hang Dong district (Chiang Mai province) [RR = 1.666; 95% CrI = 1.611–1.729], then Wiang Chai district (Chiang Rai province) [RR = 1.140; 95% CrI = 1.103–1.097], Sop Prap district (Lampang province) [RR = 1.089; 95% CrI = 1.053–1.130], Mueang Lamphun district (Lamphun province) [RR = 0.942; 95% CrI = 0.912–0.978], Den Chai district (Phrae province) [RR = 0.982; 95% CrI = 0.950–1.019], and Chun district (Phayao province) [RR = 0.972; 95% CrI = 0.941–1.009], in that order (Table 2) [see RR of lung cancer mortality for all districts in Additional file 2]. The high-risk patterns of RR were found in Chiang Mai province (Fig. 2a).

**Table 2:** The three districts with the highest risk of lung cancer mortality in each province

Province	District	RR	95% CrI
Chiang Mai			
	Hang Dong	1.666	(1.611–1.729)
	Doi Lo	1.633	(1.580–1.695)
	San Pa Tong	1.467	(1.419–1.522)
Chiang Rai			
	Wiang Chai	1.140	(1.103–1.183)
	Thoeng	1.057	(1.023–1.097)
	Phaya Mengrai	1.054	(1.020–1.094)
Lampang			
	Sop Prap	1.089	(1.053–1.130)
	Mae Mo	1.010	(0.977–1.048)
	Ko kha	0.993	(0.960–1.030)
Lamphun			
	Mueang Lamphun	0.942	(0.912–0.978)
	Ban Thi	0.800	(0.774–0.830)
	Pa Sang	0.782	(0.757–0.812)
Phrae			
	Den Chai	0.982	(0.950–1.019)
	Rong Kwang	0.943	(0.912–0.978)
	Nong Muang Khai	0.907	(0.878–0.941)
Phayao			
	Chun	0.972	(0.941–1.009)
	Phu Kamyao	0.952	(0.921–0.988)
	Dok Khamtai	0.894	(0.865–0.928)

*RR* Relative risk, *CrI* Credible interval

### The spatial analysis by gender

The DIC values from the BYM model for males and females were found to be 552.4 and 530.9, respectively. The results of the spatial analysis by genders showed that the RR of lung cancer mortality for males over the whole study area ranged from 0.549–1.555 and that of females ranged from 0.525–1.978 (Fig. 2b, 2c). The results also show that in some provinces, the districts with the highest RR of lung cancer mortality were different for males and females: the Doi Lo district for males [RR = 1.555; CrI = 1.495–1.617] and Wiang Haeng for females [RR = 1.978; CrI = 1.894–2.066] in Chiang Mai province, the Sop Prap district for males [RR = 1.126; CrI = 1.083–1.171] and the Mueang Pan district for females[RR = 1.048; CrI = 1.003–1.094] in Lampang province, the Den Chai district for males [RR = 1.102; CrI = 1.060–1.146] and the Wang Chin district for females [RR = 0.814; CrI = 0.779–0.850] in Phrae province, and the Mae Chai district for males [RR = 0.941; CrI = 0.905–0.978] and the Chun district for females [RR = 0.985; CrI = 0.943–1.029] in Phayao province. However, we found that the districts with the highest RR in Chiang Rai province (Wiang Chai for males [RR = 0.979; CrI = 0.941–1.018] and females [RR = 1.311; CrI = 1.255–1.369]) and Lamphun province (Mueang Lamphun for males [RR = 0.988; CrI = 0.950–1.027] and females [RR = 0.892; CrI = 0.854–0.931]) were the same for both genders (Table 3) [see RR of lung cancer mortality by gender for all districts in Additional file 2]. The highest risk pattern of lung cancer mortality for males was found in southern Chiang Mai (Fig. 2b) whereas the highest risk pattern for females was found in northern Chiang Mai (Fig. 2c).

**Table 3:** The three districts with the highest risk of lung cancer mortality in each province by gender

Province	Gender					
	Male			Female		
	District	RR	(95% CrI)	District	RR	(95% CrI)
Chiang Mai						
	Doi Lo	1.555	(1.495–1.617)	Wiang Haeng	1.978	(1.894–2.066)
	Hang Dong	1.452	(1.396–1.509)	Hang Dong	1.830	(1.753–1.912)
	San Pa Tong	1.424	(1.369–1.480)	Doi Lo	1.508	(1.444–1.575)
Chiang Rai						
	Wiang Chai	0.979	(0.941–1.018)	Wiang Chai	1.311	(1.255–1.369)
	Phaya Mengrai	0.963	(0.926–1.002)	Chiang Khong	1.232	(1.180–1.286)
	Thoeng	0.946	(0.910–0.984)	Thoeng	1.161	(1.111–1.212)
Lampang						
	Sop Prap	1.126	(1.083–1.171)	Mueang Pan	1.048	(1.003–1.094)
	Ko kha	1.126	(1.082–1.171)	Soem Ngam	0.922	(0.883–0.963)
	Mae Mo	1.034	(0.994–1.075)	Sop Prap	0.921	(0.881–0.961)
Lamphun						
	Mueang Lamphun	0.988	(0.950–1.027)	Mueang Lamphun	0.892	(0.854–0.931)
	Ban Thi	0.938	(0.902–0.976)	Pa Sang	0.848	(0.812–0.885)
	Wiang Nong Long	0.830	(0.798–0.863)	Wiang Nong Long	0.779	(0.746–0.814)
Phrae						
	Den Chai	1.102	(1.060–1.146)	Wang Chin	0.814	(0.779–0.850)
	Rong Kwang	1.077	(1.036–1.120)	Song	0.761	(0.728–0.795)
	Nong Muang Khai	1.009	(0.970–1.049)	Den Chai	0.752	(0.720–0.786)
Phayao						
	Mae Chai	0.941	(0.905–0.978)	Chun	0.985	(0.943–1.029)
	Chun	0.917	(0.882–0.953)	Phu Kamyao	0.953	(0.912–0.995)
	Phu Kamyao	0.912	(0.877–0.948)	Mueang Phayao	0.911	(0.872–0.951)

*RR* Relative risk, *CrI* Credible interval

## Discussion

Of the six northern Thailand provinces, the risk patterns of lung cancer mortality were the highest in the west (Chiang Mai) and lowest in the east (Phayao and Phrae) and south (Lamphun), which conforms to some of the findings of Aungkulanon et al. [4] who presented geographical distributions of cause-specific mortality (including liver cancer, lung cancer, chronic obstructive pulmonary disease, diabetes etc.). Regarding lung cancer, they found that the risk of mortality was the highest in Chiang Mai and the lowest in Phayao and Phrae, as did we. However, they found that the risk was high in Lamphun whereas it was low in our study. This disparity in the results was probably due to the differences in the design of the studies used to estimate the risk. In our study, the derived data were from people previously diagnosed with lung cancer who had died of any cause whereas their data were from the death certificates of people recorded as having died from lung cancer only. In addition, the BYM model can include unknown or unobserved risk factors that are related to the risk of lung cancer mortality [45], and thus may be more appropriate for spatial or geographical analyses in risk pattern studies.

The geographical patterns of risk of lung cancer mortality could be the result of environmental determinants. For instance, the Hang Dong and Doi Lo districts with the highest risk level (RR ≥ 1.50) are located in areas affected by high indoor radon and air pollution, which supports the hypothesis that these factors can affect the risk patterns of lung cancer mortality [24, 25, 31]. Future work should examine associations between environmental factors and lung cancer mortality.

In our study, different levels of risk of lung cancer mortality were found by geographical location, which might have been due to the geographical risk patterns in lung cancer mortality closely following those of lung cancer mortality incidence [2, 53]. Therefore, the differences in the incidences between areas might have been affected by the geographical risk patterns of mortality. The spatial effects of previous studies such as the problem of high air pollution [16, 27], a geographical location characterized by high mountains [28–30], and increasing urban growth [11, 31, 32] might have been the reason for the high risk of lung cancer mortality in Chiang Mai province. A considerable number of studies on air pollution monitoring in the northern region of Thailand [10, 14, 54] found that Chiang Mai was not the area with the highest air pollution level in this region, thus it seems that this might not be the only factor in the risk of death from lung cancer.

The analysis of the risk of lung cancer mortality by gender revealed that females were at a higher risk of lung cancer mortality than males, especially in districts within Chiang Rai province. This might have been due to the effect of the chewing of Miang (fermented wild tea leaves) and tobacco smoking (cigarettes and Khiyoh) [12, 55, 56], in that the habit of females pursuing this pastime is higher than males [57]. Moreover, the severity of the stage of lung cancer at the time of diagnosis for females was higher than for males [7]. In addition, we found no significant differences in the risk patterns of lung cancer mortality by gender in Chiang Mai province, which exhibited high RR in the west of the study area for both genders. Although females had a higher risk of lung cancer mortality than males, the development of lung cancer preventative strategies should focus on both genders due to lung cancer being a healthcare burden and a leading cause of mortality in this region [4–7].

Our results may be useful to other researchers wishing to study the environmental factors related to lung cancer and other associated diseases. For example, researchers reporting the municipal distribution of bladder cancer mortality and attempting to adjust this spatial pattern for the prevalence of smoking used the estimated RR values of lung cancer mortality as a surrogate for the prevalence of smoking using the BYM model to adjust for the RR of bladder cancer mortality [58]. Moreover, the random effects modeling with Bayesian spatial models, which represent the unknown risk factors and their estimation through the posterior distribution, could help to identify underlying causes for unknown risks.

## Conclusions

In conclusion, we found that there was a high risk of lung cancer mortality in districts within Chiang Mai province, both overall and by gender. There was distinct geographical variation in risk patterns of lung cancer mortality in Thailand. Differences could be related to differences in risk factors such as ground-based radon and air pollution. However, our study was conducted to examine the spatial pattern of the risk of lung cancer mortality only. As such, this study provides a starting point for estimating the spatial pattern of the risk of lung cancer mortality and for examining associations between geographic risk factors and lung mortality for further studies.

## Additional files

Additional file 1: Appendix 1. The OpenBugs syntax for the BYM model fitting. (DOCX 13 kb)

Additional file 2: **Table S1.** Relative risks of lung cancer mortality in each district. (DOCX 26 kb)

## Abbreviations

BYM: Besag-York-Mollié.
CrI: Credible interval
DIC: The deviance information criterion
ICD: International Classification of Diseases
PM10: Particulate matter with a diameter of smaller than 10 μm
QGIS: The Quantum Geographic Information System
RR: Relative risk
SMR: Standardized mortality ratio
