# Supplementary material for: Risk patterns of lung cancer mortality in northern Thailand
Source: BMC Public Health. 2018 Sep 24;18:1138. doi: 10.1186/s12889-018-6025-1 (PMC6154807; doi:10.1186/s12889-018-6025-1)
Supplement: Supplementary file 1 — Appendix 1. The OpenBugs syntax for the BYM model fitting. (DOCX 13 kb) [file 12889_2018_6025_MOESM1_ESM.docx]

**Appendices**

Appendix 1- the OpenBugs syntax for the BYM model fitting

***# syntax***

openBugs <- bugs(data=" ",

inits=function(),

parameters=c("kappaU","u", "kappaV","v","intercept"),

model.file=" ",debug=FALSE,

codaPkg=TRUE,

OpenBUGS.pgm=" ",

n.chains=1, n.iter=15000, n.burnin=5000,

n.thin=20, bugs.seed=2)

openBugs.summary <- read.bugs(openBugs)

***# model***

model{

# likelihoood

for(i in 1:N){

Y[i] ~ dpois(landa[i])

log(landa[i]) <- log(E[i]) + u[i] + v[i] }

# convolution-prior

for(i in 1:N)[u[i] <- uConstr[i] + intercept]

intercept ~ dflat()

uConstr[1:N] ~ car.normal(adj[], weights[], num[], kappaU)

for(k in 1:sumNumNeigh) [ weights[k] <- 1 ]

for(i in 1:N)[ v[i] ~ dnorm(0, kappaV) ]

# prior

kappaU ~ dgamma(1, .5); kappaV ~ dgamma(1, .01)

}

*#* ***Spatial structure in each district***

adj = c(

5, 6, 7, 8, 10, 13, 16, 18, 41,

3, 5, 12, 15,

2, 4, 12, 15, 17, 18,

…………

…..……..

……..…..

22, 23, 63, 69, 76, 77, 80,

74, 76, 77, 79,

74, 75, 77

),
