# Supplementary material for: Risk patterns of lung cancer mortality in northern Thailand
Source: BMC Public Health. 2018 Sep 24;18:1138. doi: 10.1186/s12889-018-6025-1 (PMC6154807; doi:10.1186/s12889-018-6025-1)
Supplement: Supplementary file 2 — Table S1. Relative risks of lung cancer mortality in each district. (DOCX 26 kb) [file 12889_2018_6025_MOESM2_ESM.docx]

**Table S1. Relative risks of lung cancer** mortality in each district.

| No. | District | RR-Overall | RR-Male | RR-Female | Province |
| --- | --- | --- | --- | --- | --- |
| 1 | Mueang Chiang Rai | 0.9231 | 0.8595 | 1.0087 | Chiang Rai |
| 2 | Khun Tan | 0.9151 | 0.9135 | 0.9352 | Chiang Rai |
| 3 | Chiang Khong | 0.9861 | 0.8066 | 1.2319 | Chiang Rai |
| 4 | Chiang Saen | 0.9297 | 0.8689 | 0.9991 | Chiang Rai |
| 5 | Thoeng | 1.0573 | 0.9461 | 1.1608 | Chiang Rai |
| 6 | Pa Daet | 0.8226 | 0.9059 | 0.7546 | Chiang Rai |
| 7 | Phan | 0.9143 | 0.9224 | 0.8942 | Chiang Rai |
| 8 | Mae Chan | 0.9063 | 0.8672 | 0.9471 | Chiang Rai |
| 9 | Mae Fa Luang | 0.7252 | 0.8343 | 0.6393 | Chiang Rai |
| 10 | Mae Suai | 0.8708 | 0.7822 | 1.0616 | Chiang Rai |
| 11 | Mae Sai | 0.9551 | 0.8721 | 1.0410 | Chiang Rai |
| 12 | Wiang Kaen | 0.9797 | 0.8593 | 1.1525 | Chiang Rai |
| 13 | Wiang Chai | 1.1400 | 0.9787 | 1.3108 | Chiang Rai |
| 14 | Wiang Pa Pao | 0.8697 | 0.8165 | 0.9814 | Chiang Rai |
| 15 | Phaya Mengrai | 1.0540 | 0.9634 | 1.1376 | Chiang Rai |
| 16 | Mae Lao | 0.8783 | 0.7786 | 1.0350 | Chiang Rai |
| 17 | Doi Luang | 0.9120 | 0.8425 | 1.0007 | Chiang Rai |
| 18 | Wiang Chiang Rung | 0.6393 | 0.6207 | 0.8482 | Chiang Rai |
| 19 | Mueang Phayao | 0.8771 | 0.8462 | 0.9107 | Phayao |
| 20 | Chun | 0.9723 | 0.9168 | 0.9850 | Phayao |
| 21 | Chiang Kham | 0.6936 | 0.6551 | 0.7846 | Phayao |
| 22 | Chiang Muan | 0.7266 | 0.7949 | 0.6615 | Phayao |
| 23 | Dok Khamtai | 0.8939 | 0.8733 | 0.8949 | Phayao |
| 24 | Pong | 0.5988 | 0.5486 | 0.7532 | Phayao |
| 25 | Mae Chai | 0.8427 | 0.9407 | 0.7169 | Phayao |
| 26 | Phu Sang | 0.7626 | 0.7574 | 0.7905 | Phayao |
| 27 | Phu Kamyao | 0.9524 | 0.9117 | 0.9527 | Phayao |
| 28 | Mueang Chiang Mai | 0.9029 | 0.9356 | 0.8805 | Chiang Mai |
| 29 | Chom Thong | 1.3315 | 1.3809 | 1.1605 | Chiang Mai |
| 30 | Chiang Dao | 1.2056 | 1.0898 | 1.3703 | Chiang Mai |
| 31 | Chai Prakan | 1.2026 | 1.0744 | 1.3450 | Chiang Mai |
| 32 | Doi Tao | 1.1541 | 1.2394 | 0.8733 | Chiang Mai |
| 33 | Doi Lo | 1.6333 | 1.5553 | 1.5082 | Chiang Mai |
| 34 | Doi Saket | 1.1741 | 1.1941 | 1.1025 | Chiang Mai |
| 35 | Fang | 1.0550 | 1.0125 | 1.1108 | Chiang Mai |
| 36 | Phrao | 1.2481 | 1.0970 | 1.4216 | Chiang Mai |
| 37 | Mae Chaem | 0.7934 | 0.8946 | 0.7432 | Chiang Mai |
| 38 | Mae Taeng | 1.2628 | 1.1111 | 1.4605 | Chiang Mai |
| 39 | Mae Rim | 1.1417 | 1.1433 | 1.1387 | Chiang Mai |
| 40 | Mae Wang | 1.2912 | 1.1592 | 1.4399 | Chiang Mai |
| No. | District | RR-Overall | RR-Male | RR-Female | Province |
| 41 | Mae Ai | 1.2186 | 1.0186 | 1.4836 | Chiang Mai |
| 42 | Mae On | 1.0647 | 0.9954 | 1.0800 | Chiang Mai |
| 43 | Wiang Haeng | 1.3573 | 0.9615 | 1.9780 | Chiang Mai |
| 44 | Samoeng | 1.0977 | 1.0638 | 1.1564 | Chiang Mai |
| 45 | San Kamphaeng | 1.0644 | 1.1336 | 0.9645 | Chiang Mai |
| 46 | San Sai | 1.2516 | 1.2910 | 1.1645 | Chiang Mai |
| 47 | San Pa Tong | 1.4668 | 1.4236 | 1.4652 | Chiang Mai |
| 48 | Saraphi | 1.2920 | 1.2953 | 1.2373 | Chiang Mai |
| 49 | Hang Dong | 1.6658 | 1.4515 | 1.8304 | Chiang Mai |
| 50 | Omkoi | 0.5844 | 0.6966 | 0.5252 | Chiang Mai |
| 51 | Hot | 1.0659 | 1.2749 | 0.7043 | Chiang Mai |
| 52 | Kanlayaniwatthana | 0.7732 | 1.0147 | 0.6071 | Chiang Mai |
| 53 | Mueang Lamphun | 0.9423 | 0.9876 | 0.8916 | Lamphun |
| 54 | Thung Hua Chang | 0.5810 | 0.6668 | 0.5961 | Lamphun |
| 55 | Ban Hong | 0.6217 | 0.7348 | 0.5655 | Lamphun |
| 56 | Pa Sang | 0.7824 | 0.7758 | 0.8478 | Lamphun |
| 57 | Mae Tha | 0.6717 | 0.7660 | 0.6278 | Lamphun |
| 58 | Li | 0.6476 | 0.7207 | 0.5978 | Lamphun |
| 59 | Ban Thi | 0.8001 | 0.9384 | 0.7332 | Lamphun |
| 60 | Wiang Nong Long | 0.7605 | 0.8296 | 0.7794 | Lamphun |
| 61 | Mueang Lampang | 0.7897 | 0.8373 | 0.7311 | Lampang |
| 62 | Ko kha | 0.9927 | 1.1257 | 0.7843 | Lampang |
| 63 | Ngao | 0.9237 | 0.9660 | 0.8365 | Lampang |
| 64 | Chae Hom | 0.7727 | 0.8493 | 0.7059 | Lampang |
| 65 | Thoen | 0.8154 | 0.8917 | 0.7062 | Lampang |
| 66 | Mae Tha | 0.7788 | 0.8887 | 0.6469 | Lampang |
| 67 | Mae Phrik | 0.7651 | 0.8574 | 0.6347 | Lampang |
| 68 | Mueang Pan | 0.9650 | 0.8888 | 1.0476 | Lampang |
| 69 | Mae Mo | 1.0100 | 1.0343 | 0.8704 | Lampang |
| 70 | Wang Nuea | 0.8497 | 0.8703 | 0.8315 | Lampang |
| 71 | Sop Prap | 1.0885 | 1.1262 | 0.9206 | Lampang |
| 72 | Soem Ngam | 0.9635 | 0.9440 | 0.9218 | Lampang |
| 73 | Hang Chat | 0.9061 | 1.0194 | 0.7347 | Lampang |
| 74 | Mueang Phrae | 0.7434 | 0.8410 | 0.6359 | Phrae |
| 75 | Den Chai | 0.9821 | 1.1025 | 0.7524 | Phrae |
| 76 | Rong Kwang | 0.9425 | 1.0771 | 0.7319 | Phrae |
| 77 | Long | 0.6960 | 0.7943 | 0.6192 | Phrae |
| 78 | Wang Chin | 0.8698 | 0.8955 | 0.8139 | Phrae |
| 79 | Song | 0.8966 | 0.9774 | 0.7608 | Phrae |
| 80 | Nong Muang Khai | 0.9072 | 1.0089 | 0.7362 | Phrae |
| 81 | Sung Men | 0.8398 | 0.9642 | 0.6726 | Phrae |
